# Physiological basis of tolerance to complete submergence in rice involves genetic factors in addition to the *SUB1* gene

**DOI:** 10.1093/aobpla/plu060

**Published:** 2014-10-03

**Authors:** Sudhanshu Singh, David J. Mackill, Abdelbagi M. Ismail

**Affiliations:** 1 International Rice Research Institute (IRRI), New Delhi, India; 2 Department of Plant Sciences, Mars, Inc., University of California, Davis, CA, USA; 3 International Rice Research Institute (IRRI), DAPO Box 7777, Metro Manila, Philippines

**Keywords:** Flash floods, rainfed lowlands, recovery of submergence, rice, submergence tolerance, *SUB1*, tillering.

## Abstract

Recurring floods in Asia cause poor crop establishment. Yields decline drastically when plants are completely submerged for a few days. Traditional rice cultivars predominate because they have acquired moderate tolerance to flooding but they carry the penalty of inherently lower grain yields. In contrast, modern high-yielding varieties are highly susceptible to flooding. Cultivars with tolerance to complete submergence were recently developed in the background of popular varieties by transferring the submergence tolerance gene *SUBMERGENCE1* (*SUB1*) from the highly tolerant Indian landrace FR13A. The present study evaluated three pairs of Sub1 near-isogenic lines (NILs) together with FR13A and two of its submergence-tolerant derivatives under field conditions to assess the survival and growth processes occurring during submergence and recovery that are associated with *SUB1*. Under control conditions, the NILs showed similar growth and biomass accumulation, indicating that *SUB1* had no apparent effects. Submergence substantially decreased biomass accumulation but with greater reduction in the genotypes lacking *SUB1*, particularly when submergence was prolonged for 17 days. When submerged, the lines lacking *SUB1* showed greater elongation and lower or negative biomass accumulation. Sub1 lines maintained higher chlorophyll concentrations during submergence and lost less non-structural carbohydrates (NSC) after submergence. This indicates that the introgression of *SUB1* resulted in better regulation of NSC during submergence and that high pre-submergence NSC is not essential for the submergence tolerance conferred by *SUB1*. During recovery, chlorophyll degradation was faster in genotypes lacking *SUB1* and any surviving plants showed poorer and delayed emergence of tillers and leaves. Sub1 lines restored new leaf and tiller production faster. During submergence, FR13A showed not only slower leaf elongation but also accumulated extra biomass and was able to recover faster than Sub1 lines. This suggests the possibility of further improvements in submergence tolerance by incorporating additional traits present in FR13A or other similar landraces.

## Abstract (precis)

Complete submergence reduces survival and yield of modern high-yielding rice cultivars. Tolerant cultivars carrying the *SUB1* locus were recently developed. We evaluated survival and growth processes occurring during submergence and recovery that are associated with *SUB1* in contrasting genotypes. Sub1 cultivars showed less reduction in shoot biomass and better regulation of non-structural carbohydrates during submergence. They produced new leaves and tillers faster during recovery. FR13A, the source of *SUB1* locus showed slower leaf elongation and recovered faster than Sub1 lines, suggesting the possibility of further improvements in submergence tolerance by incorporating additional traits from FR13A or similar landraces.

## Introduction

High rainfall during the wet season (WS), overflowing rivers and canals, or high tides often flood farmland and adversely affect productivity in large areas of South and Southeast Asia. Rice is often the only crop capable of surviving under these conditions (Setter and Laureles 1996; Pucciariello and Perata 2013) and some of the flood-prone environments are still planted with traditional rice (*Oryza sativa*) landraces that are moderately adapted to flooding. However, rice productivity in areas planted in this way is low and unstable, averaging <2.0 t ha^−1^ in rainfed lowlands and <1.5 t ha^−1^ in flood-prone areas, compared with yields of >5.0 t ha^−1^ in input-intensive irrigated systems (Ismail *et al.* 2013; Singh *et al.* 2013). This results in serious crop losses and sometimes leads to severe food shortages in flood-affected regions (Mackill *et al.* 2012; Ismail *et al.* 2013).

Most higher yielding modern rice varieties die within a week of complete submergence, making them unsuitable alternatives of traditional rice landraces. However, because of their predominantly fertile soils and freshwater resources, these flood-prone ecosystems have enormous potential for enhancing food production to help meet the ever-increasing demands for rice. Furthermore, rice is the only agricultural crop able to survive these frequently flooded environments. Developing high-yielding, stress-tolerant varieties is thus a strategic imperative that aims to provide farmers with a cost-effective option in flood-affected areas (Mackill *et al.* 2012; Ismail 2013; Septiningsih *et al.* 2013; Singh *et al.* 2013).

Worldwide, more than 20 million hectares of rice are affected by flash floods each year (IRRI 1993). Flash flooding can cause complete inundation of the entire plant for several days, often for up to 2 weeks, and usually occurs at the seedling or early vegetative stage. A few traditional rice varieties, such as FR13A, can thrive in regions affected by flash floods where modern high-yielding varieties could not survive complete submergence, but these local landraces have inherently low yields and poor grain quality even under non-flooded conditions (Mackill *et al.* 1996, 2012; Mohanty *et al.* 2000; Xu *et al.* 2006). Rice genotypes adapted to this type of flooding usually stay dormant or ‘quiescent’ when flooded to conserve their energy reserves and maintain their chlorophyll and underwater photosynthesis (Ella *et al.* 2003*a**,* *b*; Das *et al.* 2005; Nagai *et al.* 2010; Winkel *et al.* 2013).

Modern high-yielding varieties are particularly sensitive to submergence even for a few days. Their yield can be severely reduced because of high mortality, low tillering and slow recovery (Ismail *et al.* 2008; Singh *et al.* 2009, 2013). A rapid decline in the oxygen (O_2_) diffusion rate (∼10 000-fold slower) in floodwater compared with in air impedes respiration leading to an energy shortage. This is particularly severe when, in addition, photosynthesis is limited or absent, because of impeded inward diffusion of CO_2_ and shading. This results in plant death either during submergence or shortly after de-submergence (Jackson and Ram 2003; Bailey-Serres and Voesenek 2008; Licausi and Perata 2009). Ethylene accumulates in plant tissue during submergence because of both enhanced synthesis and entrapment when its diffusive escape is inhibited by water and subsequently prompts underwater leaf senescence. This effect is suppressed in tolerant cultivars such as FR13A (Jackson *et al.* 1987; Ella *et al.* 2003*b*; Jackson and Ram 2003; Fukao *et al.* 2006). Accelerated loss of chlorophyll in leaves of submerged plants is caused by ethylene (Jackson *et al.* 1987), which triggers gene expression and enzyme activity of chlorophyllase, the first enzyme involved in chlorophyll breakdown. This reduces the capacity for CO_2_ fixation during and after submergence (Sarkar *et al.* 2001; Ella *et al.* 2003*b*). Damage from the action of free radicals during submergence is also less in genotypes containing *SUB1* (Santosa *et al.* 2007) and this may contribute to a stronger recovery after submergence.

Nonstructural carbohydrates (NSC) are the prime substrates for generating energy. Complete submergence causes their rapid consumption and an initiation of protein hydrolysis (Setter *et al.* 1987). An evaluation of submergence-tolerant and submergence-intolerant rice grown under unstressed conditions revealed that the seedlings of tolerant landraces normally had 30–50 % more NSC compared with sensitive genotypes (Chaturvedi *et al.* 1996; Sarkar 1998). These NSC are utilized during submergence to supply energy for growth and maintenance metabolism (Sarkar *et al.* 1996). In contrast, Mazaredo (1981) did not notice any difference between tolerant and sensitive genotypes in carbohydrate concentration before submergence but did observe a strong correlation between submergence tolerance and residual carbohydrates after submergence. Ram *et al.* (2002) also observed that the amount of NSC contained within the dry seed or in the shoots of 10-day-old seedlings prior to submergence was not necessarily higher in submergence-tolerant types; yet these genotypes tend to lose less carbohydrate when under water and recover faster after submergence (Mazaredo and Vergara 1982; Das *et al.* 2005). This suggests a slower utilization rate when underwater or, in tolerant types, a concurrent energy supply through underwater photosynthesis (Das *et al.* 2005; Winkel *et al.* 2013, 2014). Accumulation of high carbohydrates in shoots before submergence is observed in some landraces including some submergence-tolerant and deepwater traditional varieties. However, this trait does not seem to be a pre-requisite for tolerance of complete submergence (Das *et al.* 2005).

To date, the most significant finding in flood-tolerance rice research is the identification of the *SUB1A* gene on chromosome 9, as the major determinant of submergence tolerance in FR13A and its derived progenies (Xu and Mackill 1996). Using marker-assisted backcrossing, a quantitative trait locus (QTL) containing *SUB1* was recently transferred into several popular Asian rice varieties, already possessing agronomic and quality traits preferred by farmers (Siangliw *et al.* 2003; Xu *et al.* 2006; Neeraja *et al.* 2007; Septiningsih *et al.* 2009, 2013; Singh *et al.* 2010; Thomson *et al.* 2010; Manzanilla *et al.* 2011; Mackill *et al.* 2012; Collard *et al.* 2013). By providing options for use of nutrients and other inputs to enhance yields further, these varieties have provided new opportunities for farmers in submergence-prone areas to secure higher annual productivity (Ismail 2013). Sub1-containing modern varieties are identical to the original varieties in nearly all traits (Sarkar *et al.* 2009; Singh *et al.* 2009; Mackill *et al.* 2012). Consequently, they have been extensively adopted by farmers within few years of their release (Mackill *et al.* 2012; Ismail *et al.* 2013; Singh *et al.* 2013).

Being an ethylene-response factor, *SUB1A* is induced at the transcript level by submergence (Fukao *et al.* 2006) and shows no obvious effect under other conditions. Following submergence, survival of the Sub1 lines is substantially higher than that of non-Sub1 varieties. This has been consistently reflected in a yield advantage of 1 to >3 t ha^−1^ depending on the stage at which submergence occurred, the duration of submergence and the condition of the floodwater (Das *et al.* 2009; Mackill *et al.* 2012; Ismail *et al.* 2013). Moreover, Sub1 varieties flowered and matured earlier and had better grain filling than the non-Sub1 genotypes following submergence (Sarkar *et al.* 2009; Singh *et al.* 2009; Manzanilla *et al.* 2011). Whether the survival of submergence alone is contributing to this yield increase and earliness after submergence, or whether other traits, such as those associated with suppressed production of active oxygen species underwater and earlier and faster recovery, are also regulated by the *SUB1A* allele is still not known. Other genes carried by FR13A and other tolerant cultivars (Ismail and Mackill 2013) may also play a role. Additional post-flooding responses may be associated with submergence tolerance. These could include prevention of leaf dehydration (Setter *et al.* 2010) and post-submergence upregulation of scavengers of reactive oxygen species (Ella *et al.* 2003*a*).

Earlier studies conducted on the mechanisms of submergence tolerance in rice using the highly tolerant landrace FR13A, and more recently several Sub1 introgression lines, focused largely on the survival of plants following distinct periods of inundation. But, our own unpublished observations showed that the speed at which surviving plants recover and regenerate new tillers and leaves is also important for higher grain yield. Furthermore, there exists some variation between tolerant donors (FR13A versus its derivatives) as well as between cultivars used for Sub1 introgression. Evidence for such variation came from comparing responses of pairs of Sub1 NILs versus tolerant landraces such as FR13A and its derivatives, IR40931 and IR49830. These contain submergence-tolerant QTLs in addition to the *SUB1* locus (Mackill *et al.* 1993; Nandi *et al.* 1997). Time-course variations in post-submergence recovery and growth patterns exhibited by these genotypes under control and following submergence in the field will help establish whether these traits are regulated by *SUB1* or whether additional processes are involved, which are controlled by other genes. This information will be of utmost relevance to breeding genotypes that withstand a longer duration of complete submergence than that conferred by the *SUB1* locus alone.

The present study quantified the impact of the *SUB1* QTL using pairs of NILs developed in the background of three popular rice varieties. These were compared with the original donor (FR13A) and with two of its derivatives developed by standard crossing. Variation in yield within this set of lines was published by Singh *et al.* (2009). Traits that are likely to be associated with survival and recovery and/or with grain yield are discussed here in an attempt to identify tolerance mechanisms that are distinctively associated with *SUB1A*. By difference, this could help identify traits potentially controlled by other genes or pathways that are necessary for conferring higher tolerance.

## Methods

The trials were conducted under a tropical natural field environment at the experimental station of the International Rice Research Institute (IRRI), Los Baños, Philippines. Singh *et al.* (2009) summarized information on climatic conditions and soil properties of the experimental farm, establishment of field trials and characteristics of floodwater conditions. The driest months of the year are January through to May (dry season, DS), with the rainy season persisting for the rest of the year (WS). Average annual rainfall is ∼2000 mm with an average pan evaporation of ∼1650 mm. The daytime temperature ranges between 30 and 32 °C. The physical and chemical properties of the clay-textured soils in different experimental plots (control block and deepwater ponds) were similar because the trial plots were adjacent to each other. The soil pH ranged from 7.5 to 7.7 and organic carbon ranged from 1.0 to 1.8 %. Kjeldahl N (%), available Olsen's P (mg kg^−1^), and available K (meq 100 g^−1^) ranges were 0.106–0.175, 17–23 and 1.34–1.68, respectively (Singh *et al.* 2009, 2011).

### Establishment of field trials

We evaluated the following six lines in two field experiments: Swarna-Sub1 and its recurrent parent Swarna; FR13A, the tolerant landrace from Orissa, India (the source of the *SUB1A* gene); two tolerant genotypes, IR49830-7-1-2-3 (IR49830) and IR40931-33-1-3-2 (IR40931) developed from FR13A (Mackill *et al.* 1993; Neeraja *et al.* 2007) and one variety (IR42) that is highly vulnerable to submergence. The first trial was conducted during the August 2005–January 2006 WS and then during the March–October 2006 DS to evaluate the performance of Swarna-Sub1 under both control conditions and following (i) moderate stress (12 days complete submergence) when 40–50 % of the sensitive check IR42 showed severe injury symptoms and (ii) severe stress (17 days complete submergence) when 70–80 % of IR42 showed severe injury symptoms. In a third experiment, IR64-Sub1 and Samba Mahsuri-Sub1 were compared with their recurrent parents and with a tolerant check (IR49830) and a sensitive check (IR42). These were evaluated once during the January–July 2007 DS under either controlled conditions or severe submergence stress of 17 days (until IR42 showed 70–80 % severe injury symptoms). A randomized complete block design (RCBD) with four replications was used for each experiment.

Seeds of all cultivars were sown in soil in nursery trays using three seeds per hole. Fourteen-day-old seedlings were transplanted in the field at 20 × 20-cm spacing, with two seedlings per hill, in 5 × 4 m plots. Ten extra rows of IR42 (∼250 hills) were planted on one side of the deep pond to observe the extent of damage of this sensitive check and to use this as a guide to decide when to terminate the submergence treatment. Nitrogen, phosphorus, potash and zinc were applied at 90-30-30-5 kg ha^−1^. Full phosphorus and potash and one-third of the nitrogen were added through a complete fertilizer, along with zinc as zinc sulphate heptahydrate, as basal at 1 day before transplanting. The remaining nitrogen was applied as urea in two splits, one at maximum tillering and one at panicle initiation. Plants were submerged at water depths of 1–1.25 m at 14 days after transplanting (DAT). Water depth was maintained by adding water regularly to the ponds. Following the first 7 days of submergence, 10 plants of IR42 were randomly uprooted daily from the extra rows to observe the extent of damage and to help decide on the time to terminate the submergence treatment.

### Assessment of survival and growth

The number of hills was noted 1 day prior to submergence in a marked 5.2 m^2^ area in the centre of each plot. Survival was then rated 21 days after submergence had ended by counting the surviving hills able to produce at least one new leaf from the same marked area, expressed as the percentage of the initial number of plants before submergence. Plant height (cm) of 12 randomly selected hills from each treatment was measured from the base of the stem to the tip of the longest leaf or of the panicle if longer, before and after submergence. Shoot elongation during submergence was computed as the percentage of the pre-submergence value. The whole root system of these 12 hills was washed carefully and separated. Length of the longest root (cm) was measured with a metre stick before and after submergence and root elongation was expressed as a percentage of the pre-submergence values. Twelve hills from each experimental plot were then separated into shoot and root parts and samples were freeze dried for 5 days and then weighed to determine the dry weights of separate organs.

Numbers of tillers and leaves per hill were counted from 12 randomly selected hills at different growth stages and their average values were multiplied by the number of hills m^−2^. Leaf area was measured on 12 hills using a leaf area meter (Model LI-3100 Area Meter, Licor, Inc., Lincoln, NE, USA) and the leaf area index (LAI) was calculated using the formula of Watson (1958). Twelve hills from each experimental plot were then separated into shoot and root and oven dried at 70 °C. Shoot, leaves and panicle dry weights were then determined separately. Crop growth rate (g m^−2^ day^−1^) was calculated using the formula of Brown (1984).

### Chlorophyll and NSC

Concentrations of total chlorophyll (C*a*+*b*) were determined on leaf samples harvested before and after submergence. Samples were freeze dried and cut into fine pieces. Chlorophyll concentration was determined following the method of Mackinney (1941) in acetone extracts. Readings were carried out using an UV–Visible spectrophotometer (DU-800^R^, Beckman Coulter™, Inc., Harbor Blvd, CA, USA) and the optical densities were recorded at 663, 652 and 645 nm. Chlorophyll concentrations were then calculated using the formula C*a* + *b* = 27.8 × A_652_ (Bruinsma 1963) and expressed as percentages of the dry weight of leaves. Total soluble sugar concentrations were determined on stem samples harvested before and after submergence. The harvested samples were frozen in liquid N_2_, freeze dried and ground to a fine powder and extracted using 80 % ethanol (v/v). Approximately 500 μL of the extract were then used for soluble sugar analysis after addition of 5 mL anthrone reagent, followed by measurement of absorbance at 620 nm using an UV–visible spectrophotometer (DU-800^R^, Beckman Coulter™) as described by Fales (1951). The residue remaining after soluble sugar extraction was oven dried and used for starch analysis following the method of Setter *et al.* (1989). Starch was solubilized in boiling water for 3 h with further hydrolysis using amyloglucosidase (Sigma Chemicals, St. Louis, MO, USA) and subsequently analysed for free sugars using glucose oxidase (Sigma Chemicals). Absorbance was read at 450 nm against a sample blank (reference) using an UV–visible spectrophotometer (DU-800^R^, Beckman Coulter™) as described by Kunst *et al.* (1988).

### Statistical analysis

Data were subjected to analysis of variance (ANOVA) using IRRISTAT for Windows version 4.4 (IRRI 2004). Analysis of variance was performed individually for each trial using an RCBD model with four replications. To detect the interactions between stress environments and genotypes, combined ANOVA of respective experiments was conducted. Experimental errors of each separate trial were examined for variance heterogeneity through non-significant *P*-values of Bartlett's test. Associations between parameters were examined using linear correlation and regression analysis.

## Results

### Monitoring of climatic and floodwater conditions

Meteorological data for the duration of different trials (mean values over 10 days) were obtained from the IRRI Climate Unit. Floodwater O_2_ concentration, pH, temperature and incident irradiance in the air and underwater were measured during submergence as discussed in Singh *et al.* (2009). Seasonal variation was observed in different climatic parameters before, during and after submergence. Continuous rainfall and cloudiness were experienced during most of the submergence duration in the WS. This reduced average daily sunshine hours to almost half of those experienced in the DSs. The average solar radiation (MJ m^−2^) received during the submergence period of the 2006 and 2007 DSs were, respectively, 10 and 25 % higher than that received during the 2005 WS. The minimum, maximum and mean daily temperatures during submergence in 2005 WS and 2006 DS were almost the same except that they were slightly lower in the 2007 DS. Throughout the water profile, the concentration of O_2_ was lower in the morning (0830 h; 0.130–0.140 mol m^−3^) and higher in the afternoon (1430 h; 0.170–0.180 mol m^−3^) during submergence. The dissolved O_2_ concentration in water was higher at the start of submergence but declined progressively with increasing submergence duration. In the morning, floodwater pH was a little lower (7.4–7.8) than in the afternoon (7.9–8.1) and was lower at the water surface than at lower depths. The pH also increased from 7.4 at the beginning to 8.1 at the end of the submergence period. Water temperature was slightly cooler in the morning and warmer in the afternoon. In general, water temperature ranged from 27 to 32 °C. Photosynthetically active radiation declined sharply with increasing water depth to 50 and 100 cm during submergence. About 60–70 % of the incident light was received at the water surface (2.5 cm), but it decreased to ∼40–50 % at 50-cm depth and further to 20–30 % at 100 cm. Total incident radiation measured in air above the floodwater was 25 % lower during the WS than the DS. In addition, more irradiance was impeded during submergence in the WS than in the DS where it was reduced by up to 75 % at the 50-cm depth during the WS, but only by half, even at 100 cm during the DS (Singh *et al.* 2009).

### Effect of submergence on survival and growth

**Figure 1.**
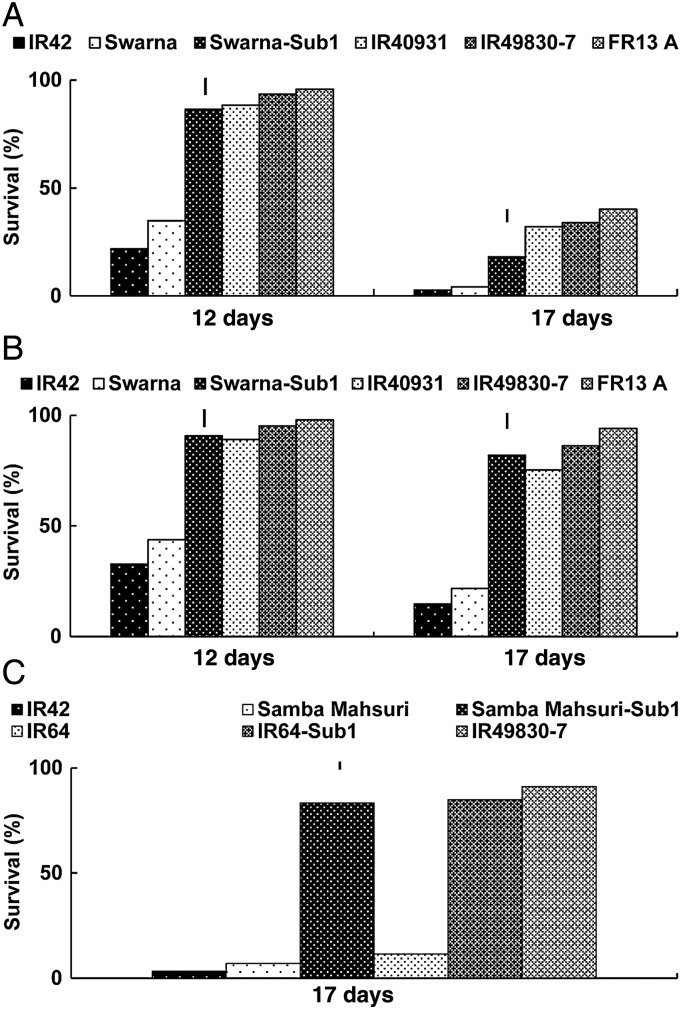
Survival of rice genotypes submerged completely for 12 or 17 days in field trials conducted during the (A) 2005 WS, (B) 2006 DS and (C) 2007 DS. Vertical lines indicate LSD_0.05_ for genotypic means within each trial.

Survival decreased substantially following submergence. When submerged for 12 and 17 days, Swarna-Sub1 showed, respectively, 2- and 4-fold greater survival than Swarna during the 2005 WS and 2006 DS. Among the tolerant genotypes, FR13A showed the highest survival, followed by IR49830 and Swarna-Sub1 (Fig. 1A and B). A similar response was observed for IR64-Sub1 and Samba Mahsuri-Sub1 introgression lines during the 2007 DS. After 17 days of submergence, the survival of Samba Mahsuri and IR64 was reduced to 6.9 and 11.4 %, while that of Samba Mahsuri-Sub1 and IR64-Sub1 was 83 and 85 %, respectively (Fig. 1C). The results clearly demonstrate the advantage of introgressing *SUB1* and its effectiveness on plant survival across variable genetic backgrounds and seasons.

Shoot elongation was similar in all genotypes under controlled conditions, but it increased progressively with the duration of submergence. The tolerant checks and Sub1 introgression lines elongated at significantly slower rates compared with the sensitive cultivars, including the recurrent parents (Table 1). FR13A, the most tolerant genotype, had the slowest underwater elongation, followed by IR49830 and the other Sub1 introgression lines. All three sensitive parental lines showed almost 1.5- to 2-fold increase in elongation under submergence compared with their Sub1 near-isogenic lines (NILs) but the Sub1 NILs showed similar elongation to that of the parents under control conditions (Table 1). Submergence-induced shoot elongation correlated negatively with plant survival (Table 2). The corresponding *r* values were −0.90 and −0.87 after 12 days of submergence in the 2005 WS and the 2006 DS. When submerged for 17 days, the *r* values were −0.91, −0.93 and −0.91 during the 2005 WS and the 2006 and 2007 DSs, respectively. This strong negative association of shoot elongation with survival clearly demonstrated the important role of *SUB1* in improving survival; its effect being closely associated with a restriction on shoot extension during submergence.

**Table 1.** Percentage shoot and root elongation of rice genotypes under control, and following 12 and 17 days of submergence. Samples were taken at 14, 28 and 33 DAT, corresponding to 0, 12 and 17 days of complete submergence. Percentage values are calculated relative to the corresponding values of plant height and root length before submergence. Ns, nonsignificant; *, **, ***, significant at *P* < 0.05, 0.01, and 0.001, respectively.

Genotypes	% Shoot elongation				% Root elongation			
	12 days		17 days		12 days		17 days	
	Control	Sub.	Control	Sub.	Control	Sub.	Control	Sub.
*2005 WS*								
IR42	93.4	114.6	118.9	154.0	39.2	10.4	89.1	1.1
Swarna	83.2	104.0	113.5	124.4	35.0	10.8	85.4	2.0
Swarna-Sub1	84.1	63.4	111.2	86.7	34.9	13.8	88.2	6.9
IR40931	107.2	60.0	145.4	73.9	48.4	14.7	90.4	11.4
IR49830	86.1	52.5	116.9	70.7	55.5	16.1	90.0	15.2
FR13A	117.7	48.1	185.3	59.0	45.5	18.3	86.0	18.4
LSD_0.05_	24.1*	30.6**	24.3***	30.3***	8.1***	2.8***	Ns	2.6***
*2006 DS*								
IR42	73.4	94.2	103.1	140.0	31.0	11.8	48.4	10.1
Swarna	77.4	102.1	113.2	136.0	31.4	12.2	59.6	10.0
Swarna-Sub1	76.8	65.2	111.9	84.3	31.5	18.5	57.5	20.1
IR40931	75.0	49.8	103.2	66.6	34.7	19.9	58.7	19.8
IR49830	56.0	37.4	80.2	54.9	30.2	20.4	55.6	22.7
FR13A	85.4	53.4	114.7	64.5	28.7	22.6	54.6	28.3
LSD_0.05_	7.8***	10.2***	11.4***	11.7***	Ns	2.1***	Ns	2.4***
*2007 DS*								
IR42	–	–	60.5	98.7	–	–	62.4	4.1
Samba Mahsuri	–	–	71.1	97.5	–	–	67.2	3.9
S. Mahsuri-Sub1	–	–	69.1	59.8	–	–	69.0	17.5
IR64	–	–	58.7	75.3	–	–	63.3	3.9
IR64-Sub1	–	–	55.4	44.9	–	–	64.6	16.5
IR49830	–	–	58.0	40.9	–	–	61.2	17.6
LSD_0.05_	–	–	Ns	8.0***	–	–	Ns	2.4***

**Table 2.** Relationship among survival rate (%), shoot and root elongation (elong; %), pre- (Bs) and post-submergence (As) stem, leaf and root dry weights (wt; g m^−2^), stem starch and sugar concentrations (con; %) and chlorophyll (*a* + *b*) concentration (chl. *a* + *b*; %) of rice genotypes after 12 and 17 days of complete submergence in field trials during the 2006 DS. *, **, ***, significant at *P* < 0.05, 0.01 and 0.001 %, respectively.

Parameters	Survival	Shoot elong	Root elong	Bs stem dry wt	As stem dry wt	Bs leaf dry wt	As leaf dry wt	Bs root dry wt	As root dry wt	Bs stem starch	As stem starch	Bs stem sugar	As stem sugar	Bs chl. (*a* + *b*)
*12 d submergence*														
Shoot elongation	−0.87***													
Root elongation	0.91***	−0.86***												
Bs stem dry wt	0.47*	−0.65***	0.60**											
As stem dry wt	0.91***	−0.86***	0.90***	0.64***										
Bs leaf dry wt	0.31	−0.57**	0.42*	0.92***	0.46*									
As leaf dry wt	0.87***	−0.84***	0.89***	0.73***	0.97***	0.52**								
Bs root dry wt	0.11	−0.41*	0.24	0.70***	0.17	0.86***	0.24							
As root dry wt	0.63**	−0.58**	0.71***	0.74***	0.82***	0.51*	0.88***	0.18						
Bs stem starch	0.23	−0.04	0.30	0.35	0.36	0.08	0.49*	−0.21	0.69***					
As stem starch	0.50*	−0.37	0.59**	0.52*	0.61**	0.22	0.72***	−0.06	0.84***	0.90***				
Bs stem sugar	0.35	−0.24	0.48*	0.53**	0.46*	0.23	0.59**	−0.04	0.74***	0.84***	0.87***			
As stem sugar	0.78***	−0.67***	0.86***	0.59**	0.85***	0.32	0.90***	0.05	0.84***	0.63***	0.84***	0.71***		
Bs chl. (*a* + *b*)	−0.0001	0.28	−0.09	−0.41*	−0.16	−0.43*	−0.16	−0.33	−0.15	0.02	−0.04	−0.07	−0.11	
As chl. (*a* + *b*)	0.82***	−0.69***	0.80***	0.61**	0.80***	0.39	0.86***	0.14	0.79***	0.55**	0.74***	0.69***	0.84***	0.02
*17 d submergence*														
Shoot elongation	−0.93***													
Root elongation	0.91***	−0.87***												
Bs stem dry wt	0.60**	−0.66***	0.65***											
As stem dry wt	0.97***	−0.95***	0.93***	0.73***										
Bs leaf dry wt	0.51*	−0.64***	0.54**	0.95***	0.64***									
As leaf dry wt	0.91***	−0.94***	0.88***	0.78***	0.98***	0.78***								
Bs root dry wt	0.19	−0.35	0.25	0.60**	0.32	0.83***	0.39							
As root dry wt	0.79***	−0.70***	0.79***	0.61**	0.85***	0.51*	0.85***	0.29						
Bs stem starch	0.39	−0.27	0.58***	0.47*	0.45*	0.23	0.38	−0.11	0.53*					
As stem starch	0.81***	−0.66***	0.88***	0.63***	0.81***	0.46*	0.73***	0.10	0.74***	0.78***				
Bs stem sugar	0.00	0.19	0.16	0.18	0.00	−0.01	−0.09	−0.21	0.07	0.74***	0.47*			
As stem sugar	0.86***	−0.83***	0.80***	0.48*	0.83***	0.39	0.78***	0.01	0.67***	0.43*	0.73***	0.04		
Bs chl. (*a* + *b*)	−0.01	0.23	−0.04	−0.28	−0.14	−0.42*	−0.25	−0.53**	0.01	0.41*	0.26	0.54**	0.18	
As chl. (*a* + *b*)	0.79***	−0.63***	0.75***	0.37	0.78***	0.24	0.61**	−0.10	0.67***	0.59**	0.82***	0.31	0.73***	0.36

Root elongation was minimal in all genotypes when submerged compared with under comparable control conditions. The tolerant genotypes showed greater root elongation than the sensitive ones (Table 1). All three Sub1 lines showed 2- to 5-fold greater increases in underwater root elongation than their recurrent parents. In contrast to shoot elongation, strong positive correlations were observed between root elongation and plant survival after submergence (Table 2). When submerged for 17 days, the correlation coefficients were 0.90, 0.91 and 0.97 during the 2005 WS and the 2006 and 2007 DSs, respectively. This positive association of survival with growth indicates the ability of the Sub1 lines to maintain carbohydrate supply to roots and aeration of roots to support their limited growth and function.

Submergence-induced inhibition of dry matter accumulation in stems was relatively higher in sensitive genotypes and especially under the severe stress of 17 days underwater (Table 3). Plants submerged during the WS showed a greater reduction in stem dry weight than plants submerged in the DS. The Sub1 lines and the tolerant checks (FR13A and IR49830) showed a significant increase in stem dry weight, even after 17 days of submergence compared with their initial weight before submergence. However, the sensitive genotypes showed either a reduction in weight or merely maintained their pre-submergence weight. The three pairs of NILs had similar stem dry weights when grown under control conditions.

**Table 3.** Stem, root and leaf dry weights and root/shoot ratios of rice genotypes before submergence (Bs) and following 17 days of submergence (As) in field trials conducted during different seasons. Samples were taken at 14 and 33 DAT, corresponding to 0 and 17 days of complete submergence. **, ***, significant at *P* < 0.01 and 0.001, respectively.

Genotypes	Stem dry weight (g m^−2^)		Leaf dry weight (g m^−2^)		Root dry weight (g m^−2^)		Root/shoot ratio	
	Bs	As	Bs	As	Bs	As	Bs	As
*2005 WS*								
IR42	2.6	1.2	2.7	0.8	0.8	0.3	0.146	0.189
Swarna	2.9	1.4	3.2	1.0	0.9	0.4	0.152	0.221
Swarna-Sub1	3.0	2.1	3.3	1.8	1.0	0.6	0.154	0.189
IR40931	3.2	2.8	3.6	2.5	1.2	0.8	0.175	0.205
IR49830	3.5	3.0	3.8	2.4	1.3	0.9	0.174	0.206
FR13A	4.0	3.4	3.9	2.8	1.1	0.8	0.136	0.160
LSD_0.05_	0.4***	0.3***	0.4***	0.3***	0.1***	0.1***	0.015**	0.017***
*2006 DS*								
IR42	3.8	3.6	3.4	1.8	1.4	1.1	0.193	0.196
Swarna	3.5	3.2	3.2	1.6	1.3	1.0	0.183	0.209
Swarna-Sub1	3.6	7.6	3.3	3.6	1.2	1.2	0.177	0.110
IR40931	4.1	8.5	3.7	5.2	1.4	1.3	0.184	0.094
IR49830	4.3	9.6	4.0	5.9	1.6	1.5	0.195	0.095
FR13A	4.4	10.4	3.8	5.9	1.4	1.6	0.168	0.098
LSD_0.05_	0.3***	0.6***	0.3**	0.5***	0.1**	0.2**	0.070***	0.014***
*2007 DS*								
IR42	3.9	3.4	3.6	1.9	2.0	1.8	0.273	0.338
Samba Mahsuri	3.5	3.3	3.3	1.2	1.5	1.7	0.225	0.358
S. Mahsuri-Sub1	3.6	6.0	3.3	2.6	1.6	2.6	0.238	0.297
IR64	4.3	4.0	4.0	1.5	2.1	2.4	0.256	0.409
IR64-Sub1	4.6	7.4	4.2	3.1	2.3	3.2	0.265	0.305
IR49830	5.1	8.1	4.8	3.7	2.5	3.0	0.265	0.251
LSD_0.05_	0.7***	1.0***	0.6***	0.5***	0.4***	0.4***	0.019**	0.042***

In general, the trends in leaf dry weight were similar to those of stem dry weight (Table 3), but the magnitude of reduction in dry weights measured immediately after submergence was greater for leaves than for stems. In contrast to stems, leaves of tolerant genotypes did not gain dry weight underwater, during the 2006 DS, and even showed a greater reduction in leaf dry weight (20–25 %) compared with the pre-submergence weights. After 17 days of submergence during the 2005 WS, leaf dry weights of sensitive genotypes decreased by up to 70 % but by only 30–40 % in tolerant genotypes. Leaf dry weights of Sub1 introgression lines measured at de-submergence were closely similar to those of the tolerant check IR49830. However, they did not differ significantly from their sensitive parents under control conditions or before submergence. In other words, these tolerant lines were capable of maintaining a stable leaf dry weight throughout 17 days of complete submergence.

The pattern in changes in root dry weight before and after submergence was similar to that of stem dry weight (Table 3). The root dry weight of sensitive genotypes either decreased or was maintained during submergence. In contrast, the tolerant checks and Sub1 lines gained root weight while submerged during the 2006 and 2007 DSs. During the 2005 WS, root dry weights were reduced in all genotypes but with more severe reductions being observed in the sensitive genotypes. Under control conditions, there was no difference in root dry weight between Sub1 introgression lines and their parents 14, 28 and 33 DAT. In general, after submergence the sensitive genotypes possessed greater root/shoot ratios than the tolerant genotypes, mainly because the reduction in their shoot biomass was much lower compared with that of the root biomass (Table 3). The Sub1 introgression lines did not differ significantly from their recurrent parents in the root/shoot ratio before submergence, but they had significantly lower root/shoot ratio following submergence mainly because they achieved relatively higher shoot growth underwater.

Post-submergence stem dry weight correlated positively and strongly with survival, with *r* values of 0.98, 0.97 and 0.91 during the 2005 WS and the 2006 and 2007 DSs, respectively, following 17 days of submergence. However, correlations of the corresponding pre-submergence values with survival were relatively low (*r* values of 0.80, 0.60 and 0.43, respectively). Similarly, survival after submergence showed a relatively closer association with post-submergence leaf dry weight than with pre-submergence leaf dry weight (Table 2). The link between survival and post-submergence leaf dry weight was stronger when the stress was severe. Survival correlated positively with post-submergence root dry weights with *r* values of 0.92, 0.79 and 0.79 after 17 days of submergence during the 2005 WS and the 2006 and 2007 DSs, respectively. There was no such association between survival and pre-submergence root dry weight. Apparently partial, albeit slow, shoot and root growth and biomass accumulation during submergence, particularly in culms and roots, are important for survival yet would not, for practical reasons, have been targeted in breeding. These data also suggest that *SUB1* did not completely hinder shoot elongation but rather slowed it to a level that can be supported by available carbohydrates generated during submergence.

### Chlorophyll and carbohydrate concentrations

**Figure 2.**
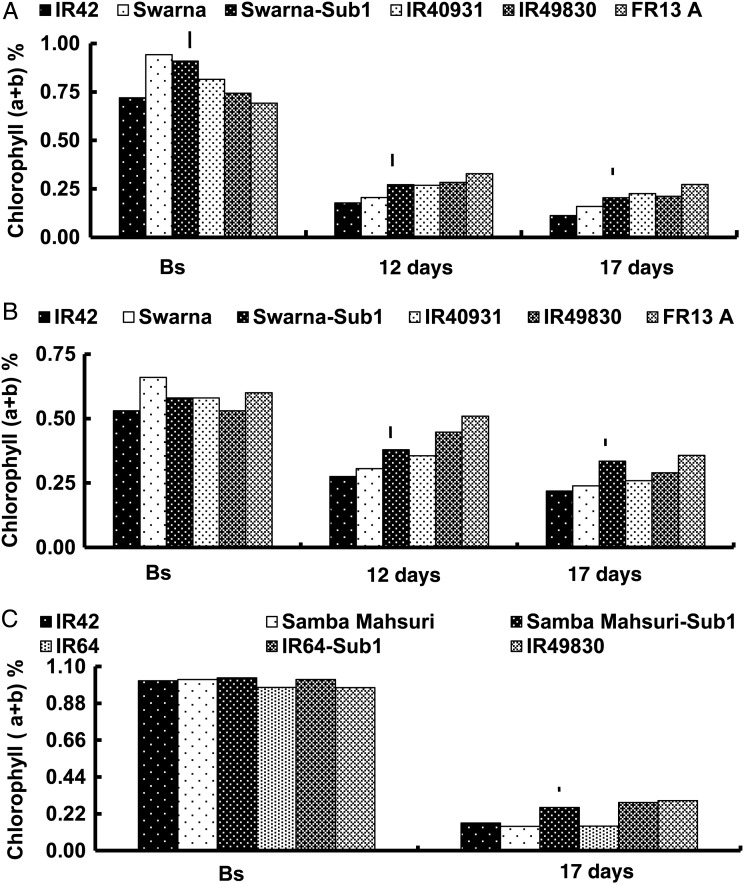
Leaf chlorophyll (*a* + *b*) concentration (%) of rice genotypes (A) before complete submergence (Bs) and after submergence of 12 or 17 days during the 2005 WS and (B) the 2006 DS; and (C) before and after complete submergence for 17 days during the 2007 DS. Vertical lines indicate LSD_0.05_ for genotypic means within each trial.

Chlorophyll concentration in leaves decreased under submergence and with increasing submergence duration from 12 to 17 days (Fig. 2). The sensitive and tolerant genotypes had similarly high leaf chlorophyll concentrations before submergence but, when submerged, the tolerant genotypes maintained more chlorophyll than the intolerant genotypes. The sensitive genotypes exhibited 10–20 % greater reduction in leaf chlorophyll concentration than did the tolerant checks and the Sub1 introgression lines. The pairs of Sub1 NILs did not differ significantly in leaf chlorophyll concentrations before submergence and under control conditions, yet concentrations were significantly higher in the Sub1 genotypes following submergence. Survival did not correlate with pre-submergence leaf chlorophyll (*a* + *b*) concentration (Table 2). However, correlations were positive with post-submergence chlorophyll (*a* + *b*) concentrations (respective *r* values of 0.76, 0.79 and 0.97 for the 2005 WS and the 2006 and 2007 DSs).

**Figure 3.**
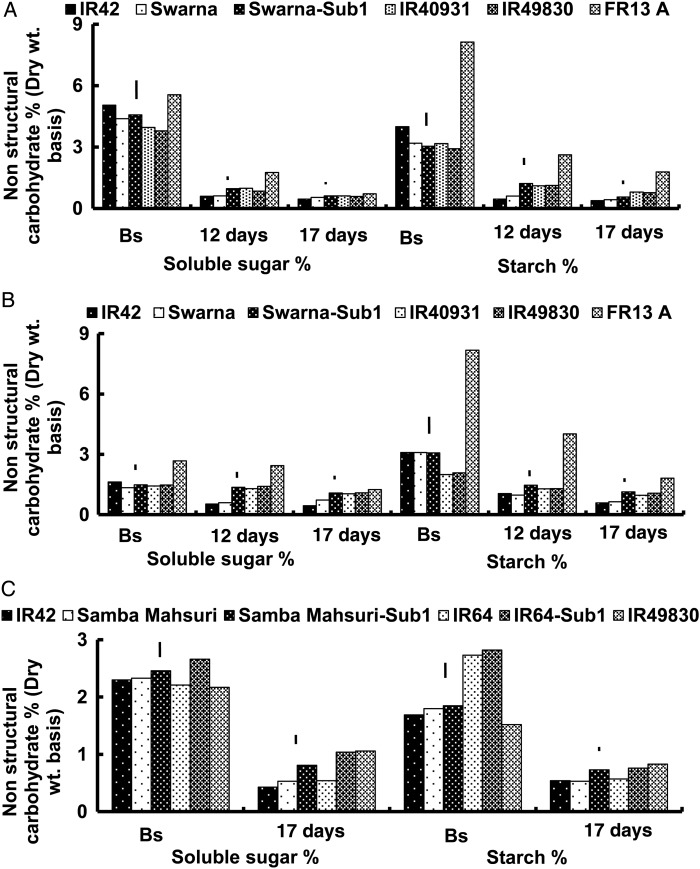
Stem soluble sugar and starch concentrations (%, dry wt basis) (A) before complete submergence (Bs) and after complete submergence for 12 or 17 days during the 2005 WS, (B) before submergence (Bs) and after submergence for 12 and 17 days during the 2006 DS, and (C) before submergence and after submergence for 17 days during the 2007 DS. Vertical lines indicate LSD_0.05_ for genotypic means within each trial.

In general, there was no specific trend in differences between tolerant and sensitive genotypes in their stem sugar and starch concentrations before submergence. The exception was FR13A, which had exceptionally high stem sugar and starch (Fig. 3). The other FR13A derivatives IR49830 and IR40931 had similar or even lower stem sugar and starch concentrations than the sensitive IR42 check. The Sub1 introgresssed lines did not differ significantly from their recurrent parents in soluble sugar and starch concentrations before submergence. These data clearly show the lack of association between submergence tolerance conferred by the *SUB1* gene and the extent of NSC stored in stems before submergence.

After submergence, the decline in carbohydrates was proportionally greater during the WS than during the DS (Fig. 3) and also with prolonged flooding of 17 days (Fig. 3B). The reduction in total soluble sugar and starch was ∼60–70 % in tolerant genotypes and ∼80–90 % in the sensitive ones. Sub1 genotypes showed 10–30 % less reduction in stem soluble sugars and 5–15 % less reduction in stem starch concentrations than their parental lines. The reduction in total soluble sugar in tolerant genotypes during the 2007 DS was ∼50–65 %, whereas the sensitive ones showed an average reduction of ∼80 % (Fig. 3C). IR49830 showed the least starch depletion, ranging from 47 to 60 % following 17 days of submergence during the 2006 and 2007 DSs and ∼70 % in the 2005 WS. All other genotypes, including FR13A, had a faster starch depletion rate than IR49830. There was no significant correlations between plant survival after 17 days of submergence and stem sugar and starch concentrations before submergence, but stronger positive correlations with concentrations after submergence (sugars: *r* values of 0.65, 0.86 and 0.87; starch *r* values of 0.79, 0.81 and 0.90, for the 2005 WS and 2006 and 2007 DSs, respectively; Table 2). Similar trends were also observed after 12 days of submergence; however, correlations with survival were generally stronger after 17 days of submergence (Table 2).

### Post-submergence recovery

**Figure 4.**
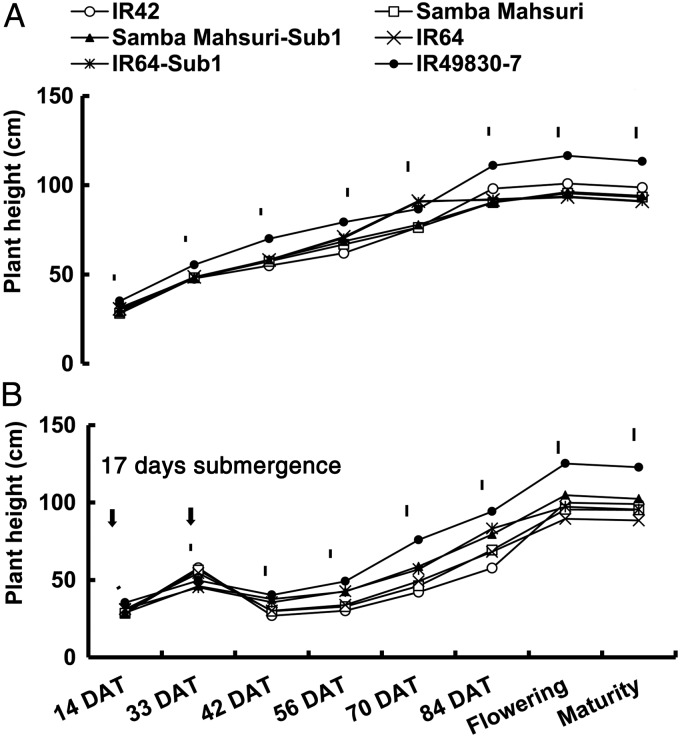
Time-course changes in shoot height of rice genotypes under (A) control and (B) following submergence for 17 days during the 2007 DS. Vertical bars above each measurement point indicated LSD_0.05_ for genotypic means. Vertical arrows indicate the start and termination of submergence.

Changes in plant height and tiller formation were monitored over time to assess differences in recovery pattern between tolerant and sensitive genotypes and also between genotypes that contain only the *SUB1* locus versus FR13A and its two derivatives, IR49830 and IR40931. Moreover, leaf and flag leaf area indices and crop growth rate were monitored at flowering and maturity, respectively. Plant height increased with time reaching its maximum near flowering. Under control conditions, plant height of the Sub1 lines was statistically similar to that of their parental lines (Fig. 4A). In submerged plots, sensitive genotypes were relatively taller at the end of treatment due to faster underwater elongation. However, their height decreased significantly immediately after submergence because of senescence and loss of most leaves. The tolerant genotypes including Sub1 lines showed faster recovery and the survivors were taller than the sensitive genotypes during early recovery stages, starting from 42 to ∼84 DAT, with differences in height narrowing towards flowering and maturity (Fig. 4B). However, the Sub1 lines, Samba Mahsuri-Sub1 and IR64-Sub1, were slightly taller than their parental lines at flowering and maturity (Fig. 4B). Almost all of the leaves of the sensitive genotypes withered during the first few days following submergence and the few surviving plants slowly initiated new leaves, while only older leaves of the tolerant genotypes senesced after submergence. They also generated new leaves faster, further contributing to their rapid recovery.

**Figure 5.**
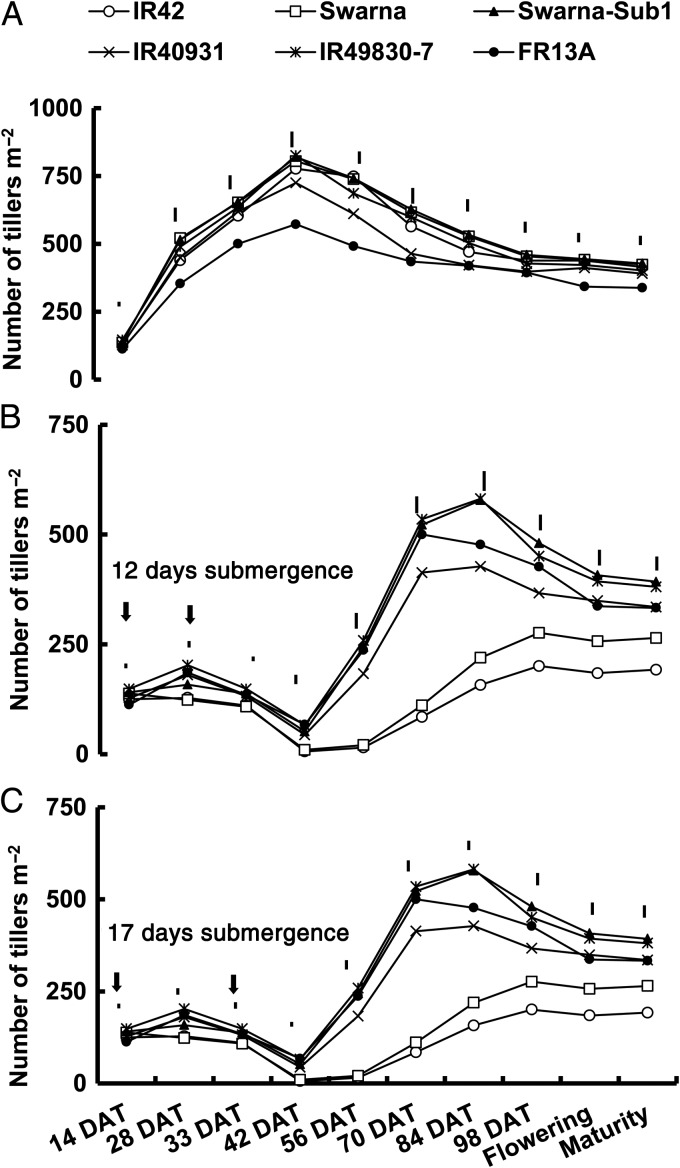
Number of tillers m^−2^ of rice genotypes under (A) control and following, (B) 12 days and (C) 17 days of complete submergence in the field during the 2006 DS. LSD_0.05_ (genotype) is shown as vertical lines above the measurement points; vertical arrows indicate the start and termination of the submergence treatment.

**Figure 6.**
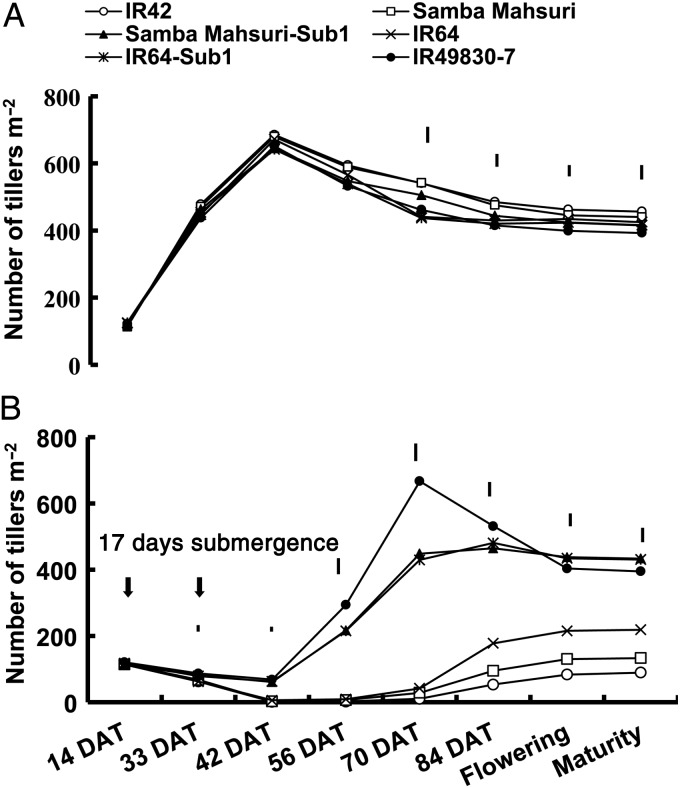
Number of tillers m^−2^ of rice genotypes under (A) control and (B) following 17 days of complete submergence in the field during the 2007 DS. LSD_0.05_ (genotype) is shown as vertical lines above the measurement points; vertical arrows indicate the start and termination of the submergence treatment.

Under control conditions, tiller number per unit land area reached its maximum in all genotypes within 33–42 DAT and then gradually decreased towards maturity (Figs 5A and 6A). Maximum tillering was reached much earlier under control conditions (33–42 DAT) than following submergence (70–84 DAT; Fig. 5). Submergence-tolerant genotypes produced substantially more tillers than did the sensitive ones after 12 and 17 days of submergence. Average tiller number per square metre after 17 days of submergence for tolerant genotypes was 450–550 m^−2^ at maximum tillering, but was less than half of this for sensitive genotypes (150–225 tillers m^−2^). A similar trend was also observed during the 2007 DS (Fig. 6A and B). None of the three Sub1 lines differed significantly from their parents under control conditions or before submergence but they had considerably more tillers m^−2^ than the parents after submergence. The tolerant checks FR13A and IR49830 maintained more tillers m^−2^ than intolerant lines and a similar number to that under control treatment, even after 17 days of submergence. All tolerant genotypes initiated tillering much earlier than the sensitive genotypes and maintained faster rates of tiller production until ∼70 DAT. The sensitive genotypes started producing new tillers at least 2 weeks later than this and continued to do so at much slower rates to reach maximum tillering just before flowering. Most tillers developed after ∼70 DAT failed to produce panicles and sometimes senesced prematurely later in the season.

No differences in LAI and FLAI were observed within Sub1 NILs at flowering under control conditions (Table 4). However, significant reductions in LAI and FLAI were noted in sensitive genotypes following submergence. The LAI and FLAI of the tolerant genotypes under control and submerged conditions were statistically similar following 12 days of submergence during the 2005 WS and after both 12 and 17 days of submergence in 2006 and 2007 DSs. However, prolonged submergence for 17 days caused significant reduction in total and flag leaf areas of the tolerant genotypes during the 2006 DS. The Sub1 lines maintained significantly higher LAI and FLAI at flowering than their parental genotypes both after 12 and 17 days of submergence. Compared with the control, reductions in LAI and FLAI in Swarna and Swarna-Sub1 lines were 41 and 7 % and 35 and 5 %, respectively, after 12 days of submergence in the 2005 WS and 21 and 4 % and 28 and 7 % in the 2006 DS. When submerged for 17 days, Swarna and Swarna-Sub1 lines, respectively, showed 50 and 7 % reductions in LAI and 55 and 13 % reductions in FLAI during the 2006 DS. During the 2007 DS, Samba Mahsuri and IR64 showed 70 and 68 % reductions in LAI and 68 and 61 % in FLAI, respectively, when submerged for 17 days, whereas their Sub1 versions showed slight increases in LAI and FLAI compared with those under the control treatment. These data showed that Sub1 genotypes recovered their leaf area much faster and reached that of the control conditions following 12 or 17 days of submergence by the time they flowered.

**Table 4.** Total (LAI) and flag leaf area indices (FLAI) calculated at flowering and CGR at maturity of rice genotypes under control, and following 12 or 17 days of complete submergence in field trials during different seasons. *, **, ***, significant at *P* < 0.05, 0.01 and 0.001, respectively.

Genotypes	LAI			FLAI			CGR (g m^−2^ day^−1^)		
	Control	12 days	17 days	Control	12 days	17 days	Control	12 days	17 days
*2005 WS*									
IR42	3.88	1.93	–	0.83	0.39	–	10.4	3.0	0.7
Swarna	3.77	2.22	–	0.80	0.52	–	9.9	3.5	1.0
Swarna-Sub1	3.80	3.54	–	0.81	0.77	–	10.2	6.3	2.6
IR40931	4.10	3.74	–	0.85	0.81	–	12.3	7.7	4.1
IR49830	4.22	4.04	–	0.93	0.89	–	11.3	7.0	3.5
FR13A	3.78	3.65	–	0.80	0.79	–	10.6	7.6	4.5
LSD_0.05_	0.33*	0.4***	–	0.08*	0.09***	–	0.9**	0.6***	0.6***
*2006 DS*									
IR42	4.50	3.21	1.87	1.10	0.68	0.41	11.3	6.1	3.5
Swarna	4.79	3.77	2.42	1.17	0.84	0.52	10.6	6.7	4.3
Swarna-Sub1	4.80	4.58	4.44	1.18	1.10	1.02	10.8	9.0	7.5
IR40931	4.79	4.63	4.09	1.13	1.04	0.87	12.6	10.0	7.3
IR49830	5.08	4.95	4.64	1.21	1.18	1.08	11.7	9.7	7.8
FR13A	4.85	4.82	4.82	0.65	0.64	0.63	7.6	7.3	6.9
LSD_0.05_	0.30*	0.73**	0.66***	0.08**	0.14***	0.15***	0.8***	0.9***	0.7***
*2007 DS*									
IR42	3.65	–	0.59	0.69	–	0.12	11.8	–	0.8
Samba Mahsuri	3.60	–	1.08	0.73	–	0.23	10.5	–	1.2
S. Mahsuri-Sub1	3.53	–	3.74	0.72	–	0.78	11.3	–	8.6
IR64	3.41	–	1.33	0.70	–	0.27	11.9	–	2.3
IR64-Sub1	3.38	–	3.46	0.68	–	0.77	12.0	–	8.7
IR49830	3.84	–	4.08	0.82	–	0.84	13.3	–	10.7
LSD_0.05_	0.23*	–	0.22***	0.06**	–	0.07***	1.0**	–	0.7***

Submergence reduced crop growth rates (CGRs) in all genotypes, with greater reduction in the sensitive genotypes and when submergence was increased from 12 to 17 days (Table 4). Crop growth rates of the three Sub1 lines under control conditions were similar to those of their recurrent parents but were significantly higher than the recurrent parents when submerged. Compared with the non-submerged controls, reduction in CGRs in Swarna and Swarna-Sub1 lines after 12 days of submergence were, respectively, 65 and 38 % in the 2005 WS and 37 and 17 % in the 2006 DS. However, these reductions were greater after the longer 17-day submergence, where the CGRs of Swarna and Swarna-Sub1 decreased by 90 and 75 %, respectively, in the 2005 WS and by 59 and 31 % in the 2006 DS. Similarly, the CGRs of Samba Mahsuri and IR64 were decreased by 89 and 81 % after 17 days of submergence during the 2007 DS, while those of their Sub1 counterparts were decreased only by 24 and 28 % after 17 days of submergence (Table 4). These data also show that submergence-tolerant genotypes, including the Sub1 lines, grew faster than the sensitive genotypes during the post-submergence recovery phase.

Post-submergence growth attributes correlated positively and significantly (*r* < 0.01) with survival. For example, following 17 days of submergence, the correlation with tiller number at maturity was positive and statistically significant (*r* values of 0.57, 0.89 and 0.97 for the 2005 WS and 2006 and 2007 DSs, respectively). Leaf area index and FLAI at flowering also correlated positively with survival following 17 days of submergence (*r* = 0.97 and 0.75 for the 2006 DS and 0.99 for both for the 2007 DS). Similarly, correlations between survival and CGR at maturity were significant with the corresponding *r* values of 0.51, 0.95 and 0.99 following 17 days of submergence for the 2005 WS and 2006 and 2007 DSs, respectively. Apparently, genotypes with higher survival also recovered faster and seemingly reflecting the dependence of recovery rate on the processes occurring during submergence.

Plant survival is an important determinant of grain yield (see Singh *et al.* 2009 for grain yield) as reflected by the positive correlations during the three seasons, with the corresponding *r* values of 0.54, 0.59 and 0.99 following 17 days of submergence. Moreover, correlations between grain yield and survival rates seem equally important as correlations between grain yield and attributes such as tillering (*r* values of 0.98, 0.84 and 0.95 for 17 days submergence), LAI and FLAI (*r* = 0.61 and 0.93 for 2006 DS, 0.99 and 0.99 for 2007 DS for 17 days submergence trials) and CGR at maturity (respective *r* values of 0.96, 0.77 and 0.99 for 17 days submergence trials). These data clearly show that survival after submergence, as well as the ability to grow and recover faster after the water recedes, are functionally related and are important determinants of yield in flood-prone areas.

## Discussion

Deployment of the *SUB1A* gene through breeding has been highly effective in reducing yield losses in flash-flood areas by conferring yield advantages of 2- to 5-fold following 12–17 days of complete submergence (Singh *et al.* 2009; 2013; Iftekharuddaula *et al.* 2011; Dar *et al.* 2013). Several studies have reported no undesirable effects on quantitative indices of grain quality, yield and yield attributes when *SUB1* was introgressed into high-yielding popular varieties (Singh *et al.* 2009; Jantaboon *et al.* 2011; Bailey-Serres *et al.* 2012; Dar *et al.* 2013). However, the effects of *SUB1* on plant growth during and after submergence and on recovery rates in different genetic backgrounds and following different durations of submergence have not been thoroughly studied up until now.

### Assessment of tolerance to submergence

The introgression of *SUB1* enhanced the survival rate of rice genotypes several folds compared with the sensitive genotypes and parental lines (Fig. 1). Similar findings were also reported earlier (Siangliw *et al.* 2003; Sarkar *et al.* 2006, 2009; Xu *et al.* 2006; Fukao and Bailey-Serres 2008; Bailey-Serres *et al.* 2010; Nagai *et al.* 2010; Sarkar and Bhattacharjee 2012). Higher shoot elongation (Table 1) during submergence was associated with intolerance and slow underwater elongation was considered desirable for submergence tolerance (Mallik *et al.* 1995*a*, *b*; Setter and Laureles 1996; Jackson and Ram 2003). Rice genotypes that elongate only slowly underwater (Sub1 types) are suitable for cultivation in flash-flood-prone areas, whereas genotypes that elongate faster during flooding are more useful for semi-deep and deepwater areas where floodwater stagnates in the field for durations of several weeks to months (Bailey-Serres and Voesenek 2008; Voesenek and Bailey-Serres 2009; Chen *et al.* 2011; Luo *et al.* 2011, Sarkar and Bhattacharjee 2012; Vergara *et al.* 2014). Recently, it has been established that *SUB1* rice cultivars survive flooding by minimizing ethylene-promoted elongation underwater. This elongation is GA-dependent and its suppression by *SUB1* is mediated by enhancement of (i) GA repressors *SLR1* and *SLR2*, which limit GA responsiveness (Fukao and Bailey-Serres 2008; Bailey-Serres *et al.* 2010) and (ii) GA catabolism mediated by differential regulation of genes associated with brassinosteroid synthesis and the induction of a GA catabolism gene (Schmitz *et al.* 2013).

In contrast to shoot elongation, the tolerant genotypes had greater root elongation than the sensitive ones (Table 1), suggesting that continued root growth and function contributes to enhanced survival under submergence and may indicate root aeration is sufficient in tolerant genotypes (Colmer *et al.* 2014). The differential root elongation patterns in different genotypes, under different submergence durations and seasons, indicate the existence of sufficient genetic variability in root sensitivity to submergence in rice. This potentially very important trait has not been sufficiently investigated hitherto but should prove useful in breeding for higher tolerance of submergence and other abiotic stresses. Singh *et al.* (2001) also observed greater root elongation in tolerant genotypes than in sensitive ones along with more adverse effects on root volume and porosity in intolerant genotypes.

Submergence-induced inhibition of dry matter accumulation was relatively higher in sensitive genotypes and under more severe stress (17 days), as shown by comparing the biomass of stems, roots and leaves before and after submergence (Table 3). Plants submerged during the DS showed less reduction in stem and leaf dry weights than plants submerged in the WS and, during the DS, tolerant genotypes even showed a slight increase in dry weight immediately after submergence compared with their dry weight before submergence. This emphasizes the likely importance of underwater photosynthesis for survival with better underwater irradiance at depth during the DS (Winkel *et al.* 2013). Total incident radiation measured in air above the floodwater was 25 % lower during the WS than the DS and more irradiance was impeded during submergence in the WS than in the DS. Survival showed relatively stronger and positive association with post-submergence stem, leaf and root dry weights than with their corresponding pre-submergence dry weight values (Table 2) and the more severe the stress, the stronger the correlation. The reduction in dry weights of stems, leaves and roots of the sensitive genotypes under submergence could be due to death and decay of tissues besides restricted concurrent underwater photosynthesis (Winkel *et al.* 2013).

Ability of the tolerant genotypes to maintain higher chlorophyll during submergence (Fig. 2) coupled with the positive correlations of survival with post-submergence leaf chlorophyll concentration (*r* = 0.82; Table 2) indicated that chlorophyll retention during and after submergence is critical for survival since it ensures underwater photosynthesis and faster recovery after the water recedes (Sarkar *et al.* 1996; Krishnan *et al.* 1999; Ella and Ismail 2006). Fukao *et al.* (2006) found that M202-Sub1 maintained higher chlorophyll than did M202 from day 6 of submergence and Sarkar *et al.* (2006) reported that 10 days of complete submergence caused greater reduction in leaf total chlorophyll concentration in Swarna than in Swarna-Sub1. Furthermore, blocking responses to ethylene were found to reduce chlorosis of leaves during submergence (Jackson *et al.* 1987; Ella *et al.* 2003*b*). Our recent studies using Swarna and Swarna-Sub1 suggested that *SUB1* is involved in chlorophyll protection during submergence, but this protection is not necessarily associated with higher or prolonged underwater photosynthesis, with the latter more likely associated with better maintenance of leaf gas films evident in FR13A (Winkel *et al.* 2014).

No specific trends of differences between tolerant and sensitive genotypes were observed in NSC concentrations prior to submergence, except in FR13A, which had much higher stem starch (Fig. 3) even in 14-day-old seedlings. These findings contrast with several previous studies that highlighted the importance of pre-submergence stem carbohydrates in submergence tolerance. However, most of these studies used FR13A as the tolerant check. The tolerant cultivars and also the Sub1 introgression lines showed smaller reductions in stem sugar and starch concentration than the sensitive IR42 and the other sensitive parents when submerged. Moreover, the most tolerant lines, IR49830 and FR13A, showed the least starch depletion. The submergence-tolerant genotypes, having relatively higher pre-submergence carbohydrates (FR13A) or less underwater carbohydrates depletion (IR49830), were probably able to maintain residual starch concentrations above the minimum threshold required for recovery after submergence (Fig. 3). This explains the invariably outstanding performance of FR13A and IR49830, even under the most severe submergence treatment stress during both seasons of field trials. A strong positive correlation was also observed between survival and concentrations of sugar and starch remaining in the stems after submergence ended (*r* = 0.81; Table 2) but not with that before submergence. Genetic differences in tolerance of submergence were, therefore, not necessarily associated with the initial carbohydrate status before submergence but rather with the ability to sustain a higher level during submergence (Mazaredo 1981; Mazaredo and Vergara 1982; Ram *et al.* 2002; Das *et al.* 2005; Sarkar *et al.* 2009; Gautam *et al.* 2014). The cultivars that are able to maintain higher NSC at the end of submergence develop new leaves more quickly and accumulate greater biomass during recovery (Panda *et al.* 2008; Sarkar and Bhattacharjee 2012). The three Sub1 introgression lines had a similar pre-submergence NSC to that of their recurrent parents. But after submergence, they displayed significantly less reduction in NSC (Fig. 3). Therefore, *SUB1* introgression does not change the basic carbohydrate content of the new lines, but instead regulates its maintenance and utilization during submergence.

High initial carbohydrate concentration in stems was also reported before in traditional rice landraces adapted to deepwater and floating rice areas, which is considered essential for accelerated elongation (Das *et al.* 2005, 2009). Sub1 lines lack this capacity, but the *SUB1A* donor FR13A, which is more tolerant than the Sub1 lines, possessed this capacity, and also possessed additional minor QTLs associated with tolerance (Nandi *et al.* 1997). Submergence tolerance of the Sub1 varieties could, therefore, be further enhanced by improving their capacity to store more NSC before submergence, possibly via incorporating other QTLs responsible for this trait from FR13A or other landraces.

### Post-submergence recovery

Sub1 lines remain shorter when submerged; however, they recover faster and became slightly taller than sensitive genotypes at maturity (Fig. 4). Faster recovery of tolerant genotypes was also associated with a shorter delay in flowering and maturity (Singh *et al.* 2009). Tolerant genotypes also produced significantly more tillers (Figs 5 and 6) per unit area than the sensitive ones after submergence. Shoots of the intolerant genotypes usually senesced and degraded with time when submerged and surviving plants recovered more slowly as shown by slower shoot elongation and leaf and tiller formation. Lodging was also higher in sensitive genotypes due to weaker culms. Tillering could be adversely affected as a result of the rapid increase in plant height during submergence (Reddy and Mittra 1985). Hanada *et al.* (1990) suggested that lack of oxygen for respiration or accumulation of ethylene might inhibit tiller bud formation and growth. During the recovery phase, the number of tillers per unit area at maturity correlated positively with survival. None of the three Sub1 lines differed significantly from their parents under control conditions, but they produced more tillers per unit area following submergence. The high mortality during submergence reduced competition and allowed for the growth of more tillers per hill during recovery, particularly in the sensitive genotypes. However, the loss in tillers per unit area could not be compensated for in the sensitive genotypes because of the drastic decrease (up to 98 %) in survival. The sensitive genotypes IR42 and the recurrent parents produced higher proportions of late tillers, which usually failed to reach maturity. Reddy *et al.* (1985) also observed a significant reduction in tiller number in rice genotypes submerged at early and active tillering stages.

Sub1 lines had the same LAI and FLAI as that of their parents under control conditions but had significantly greater LAI and FLAI following submergence (Table 4). The LAI and FLAI of the tolerant genotypes did not show major reductions following submergence, suggesting that they could recover their original LAI after flash floods. The CGR at maturity was similar in Sub1 NILs under control conditions, but significantly less in the sensitive genotypes following submergence, reflecting their slower recovery. According to Fukao *et al.* (2006), introgression of the *SUB1* locus into M202 is sufficient to restore leaf emergence fully and also the ability to resume apical meristem development upon de-submergence. They also reported that all M202-Sub1 plants produced new leaves during recovery compared with only 32 % of M202 plants.

Post-submergence growth traits such as tillers m^−2^, LAI and FLAI at flowering, and CGR at maturity correlated positively and significantly (*r* < 0.01) with survival (respective *r* values of 0.89, 0.97, 0.75 and 0.95 following 17 days submergence in the 2006 DS) and with grain yield (respective *r* values of 0.84, 0.61, 0.93 and 0.77 following 17 days submergence in the 2006 DS). These correlations between yield and post-submergence growth traits were as strong as the correlations of grain yield with yield attributes, such as panicles m^−2^, grain-filling percentage and harvest index following submergence (Singh *et al.* 2009). Clearly, genotypes having faster post-submergence growth yielded more. Sarkar and Bhattacharjee (2012) also observed cultivars with *SUB1* (Swarna-Sub1, IR64-Sub1 and Samba Mahsuri-Sub1) maintained greater biomass at the end of submergence, while resuming faster growth during recovery than their respective recurrent parents. Prompt resumption of growth following submergence is a desirable trait as it supports production of new photosynthesizing shoot biomass (Panda *et al.* 2008) and earlier tillers, both essential for higher yields. Obviously, tolerant genotypes including Sub1 lines recovered faster in terms of higher biomass, larger leaf area and more reproductive tillers. That FR13A and its derivatives maintained faster growth after submergence suggests the possibility of improving the recovery rate of the current Sub1 varieties, with further incremental increase in yield and earlier harvest. However, variation in recovery characteristics within rice germplasm has yet to be explored but would repay close attention.

The slower recovery of the intolerant genotypes could also be associated with post-flooding processes. Symptoms of injury in the sensitive genotypes are normally not evident immediately after de-submergence, but they develop progressively during post-submergence as a consequence of the damage caused by reactive oxygen species (Hunter *et al.* 1983; Monk *et al.* 1989; Gutteridge and Halliwell 1990; Crawford 1992; Ella *et al.* 2003*a*). *SUB1* might also aid recovery from submergence as evident in the findings that the tolerant genotype M202-Sub1 displayed greater upregulation of mRNAs encoding antioxidant enzymes during submergence (Jung *et al.* 2010; Mustroph and Bailey-Serres 2010; Bailey-Serres *et al.* 2012) and showed less oxidative damage upon de-submergence than M202 (Fukao *et al.* 2011). The submergence-tolerant cultivars must have acquired more efficient protective system to suppress the level of active oxygen species and lower the extent of lipid peroxidation upon exposure to air (Kawano *et al.* 2002; Ella *et al.* 2003*a*), thus showing early recovery and faster growth. Setter *et al.* (2010) also reported a large decrease in hydraulic conductivity in leaves of the sensitive variety IR42 associated with subsequent wilting and desiccation of leaves after submergence although the role of this process in submergence tolerance and recovery warrants further studies. The faster recovery observed in the tolerant rice genotypes could, therefore, be a consequence of multiple events occurring pre- and/or post-submergence.

## Conclusions

The physiological responses of Sub1 NILs to complete submergence in the field confirmed the effectiveness of *SUB1* in substantially improving the survival of submergence as well as its role in enhancing post-submergence recovery. However, variations between the tolerant donors (FR13A versus its derivatives) and the Sub1 lines clearly exist indicating that additional minor QTLs (besides *SUB1*) identified in FR13A by Nandi *et al.* (1997) and in genotypes such as IR72 by Septiningsih *et al.* (2012) could be used to further enhance tolerance of the Sub1 varieties. Recently, flood durations exceeding 20 days have been witnessed, which is beyond the tolerance provided by the *SUB1* gene.

Tolerant genotypes, including Sub1 lines, could survive complete submergence mainly because, unlike intolerant lines, they elongate very little underwater. This conserves reserve energy resources for maintenance metabolism and for use during recovery. Data on chlorophyll retention and increase in biomass during submergence suggest that tolerant varieties are also capable of CO_2_ assimilation during submergence. These tolerant genotypes also vary in pre-submergence NSC stored in their culms, with traditional landraces such as FR13A having much higher concentrations but with no obvious association with the *SUB1* gene. Genetic variation in these attributes and contribution to survival and recovery in rice await further studies. The strong associations among survival, recovery rate and grain yield point to several processes taking place during inundation and post-submergence that influence survival and growth in addition to the curtailing of shoot elongation. These include variation in underwater photosynthesis and associated traits together with the ability for continued root growth and function during submergence in addition to early tiller and leaf area formation during recovery. Results presented here provide initial evidence that potentially useful genetic variation in some of these attributes exists between Sub1 lines and FR13A. Both the survival of submergence and the ability to recover and grow faster after the water recedes are important determinants of yield in flood-prone areas. Both attributes should be considered when breeding the next generation of varieties with higher tolerance than that attainable merely by introgressing the *SUB1* gene alone.

## Source of Funding

The research was supported, in part, by the German Federal Ministry for Economic Cooperation and Development (BMZ).

## Contributions by the Authors

S.S., A.M.I. and D.J.M. conceived the study and designed the experiments. S.S. carried out the experiment under the supervision of A.M.I. for his PhD research work and both drafted the manuscript. All authors checked and approved the final draft.

## Conflicts of Interest Statement

None declared.
